# Supplementary material for: Plasmid-mediated antibiotic resistance among uropathogens in primigravid women—Hyderabad, India
Source: PLoS One. 2020 May 8;15(5):e0232710. doi: 10.1371/journal.pone.0232710 (PMC7209122; doi:10.1371/journal.pone.0232710)
Supplement: Attachment — Submitted filename: Comments on uropathogens AMR.docx [file pone.0232710.s002.docx]

**Comments on High levels of plasmid-mediated antibiotic resistance among uropathogens in primigravid women – Hyderabad, India:**

The above study was carried out with the aim of to evaluate the prevalence of plasmid-mediated-quinolone-resistance (PMQR) and extended-spectrum beta lactamase- (ESBL) producing gram-negative organisms among primigravid women with bacteriuria. This has been done to uncover the epidemiology of PMQR and ESBL 84 genes in gram-negative bacilli among primigravid women having urinary infections. Comments are given below:

- In the methodology its mentioned survey. What kind of surveys were carried out among primigravid and what information was collected details needs to be included.
- During the discussion it has been mentioned that molecular epidemiology ESBL and quinolone producing gram negative drug resistant isolates in primigravid during first visit of primigravid antenatal clinic has been described in this manuscript. However that has not been the aim of the study.
- In conclusion also study emphasises on high prevalence of drug resistant to ESBL and quinolone in community acquired UTI. Which is not included in the aims.
- In the study there is no molecular epidemiology data provided so as to corroborate with discussion as mentioned on the line no 201 to 204.
- More over its only drug resistance study using different methods including molecular levels and gene detection.
- As the study has not been compared with any other groups or with previous year data title of the study is deceptive. Needs to be corrected.

Clarification on above points may be provided and necessary changes may be made before it is reviewed again. In the present form manuscript is not suitable for publication.
