# Supplementary material for: Plasmid-mediated antibiotic resistance among uropathogens in primigravid women—Hyderabad, India
Source: PLoS One. 2020 May 8;15(5):e0232710. doi: 10.1371/journal.pone.0232710 (PMC7209122; doi:10.1371/journal.pone.0232710)
Supplement: Attachment — Submitted filename: revised comments on uropathogen amr.docx [file pone.0232710.s003.docx]

**Comments on revised :High levels of plasmid-mediated antibiotic resistance among uropathogens in primigravid women – Hyderabad, India:**

The study aim was to evaluate the prevalence of plasmid-mediated-quinolone-resistance (PMQR) and extended-spectrum beta lactamase- (ESBL) producing gram-negative organisms among primigravid women with bacteriuria. This has been done to uncover the epidemiology of PMQR and ESBL 84 genes in gram-negative bacilli among primigravid women having urinary infections. Manuscript has been revised incorporating changes as per comments given earlier. However some queries are still not addressed. Specially the one The above study was carried out with the aim of to evaluate the prevalence of plasmid-mediated-quinolone-resistance (PMQR) and extended-spectrum beta lactamase- (ESBL) producing gram-negative organisms among primigravid women with bacteriuria. This has been done to uncover the epidemiology of PMQR and ESBL 84 genes in gram-negative bacilli among primigravid women having urinary infections. Comments are given below:

lines no 203----in the beginning of discussion regarding molecular epidemiology. Discussion first three lines may be deleted. Discussion can be started with the aims as given in the abstract.

In the discussion data to show high prevalence may be compared with Indian studies also showing high levels of resistance. Merely giving references is not sufficient. At least compare the data and show it’s high.

After incorporating the changes manuscript can be accepted.
